# From lithotroph- to organotroph-dominant: directional shift of microbial community in sulphidic tailings during phytostabilization

**DOI:** 10.1038/srep12978

**Published:** 2015-08-13

**Authors:** Xiaofang Li, Philip L. Bond, Joy D. Van Nostrand, Jizhong Zhou, Longbin Huang

**Affiliations:** 1 Centre for Mined Land Rehabilitation, Sustainable Minerals Institute, The University of Queensland, QLD 4072, Australia; 2 Advanced Water Management Centre, The University of Queensland, QLD 4072, Australia; 3 Institute for Environmental Genomics and Department of Microbiology and Plant Biology, University of Oklahoma, Norman, OK 73019; 4 Earth Science Division, Lawrence Berkeley National Laboratory, Berkeley, CA, USA 94720; 5 State Key Joint Laboratory of Environment Simulation and Pollution Control, School of Environment, Tsinghua University, Beijing 100084, China

## Abstract

Engineering microbial diversity to enhance soil functions may improve the success of direct revegetation in sulphidic mine tailings. Therefore, it is essential to explore how remediation and initial plant establishment can alter microbial communities, and, which edaphic factors control these changes under field conditions. A long-term revegetation trial was established at a Pb-Zn-Cu tailings impoundment in northwest Queensland. The control and amended and/or revegetated treatments were sampled from the 3-year-old trial. In total, 24 samples were examined using pyrosequencing of 16S rRNA genes and various chemical properties. The results showed that the microbial diversity was positively controlled by soil soluble Si and negatively controlled by soluble S, total Fe and total As, implying that pyrite weathering posed a substantial stress on microbial development in the tailings. All treatments were dominated by typical extremophiles and lithotrophs, typically *Truepera*, *Thiobacillus*, *Rubrobacter*; significant increases in microbial diversity, biomass and frequency of organotrophic genera (typically *Nocardioides* and *Altererythrobacter*) were detected in the revegetated and amended treatment. We concluded that appropriate phytostabilization options have the potential to drive the microbial diversity and community structure in the tailings toward those of natural soils, however, inherent environmental stressors may limit such changes.

Sulphidic base metal tailings represent a typical anthropogenic extreme environment, as indicated by the much lower diversity of dwelling microbial communities relative to natural soils. During tailings phytostabilization, which aims to stabilize surface tailings through the establishment of a sustainable plant cover^1^, the microbial diversity and community structure are expected to be shifted by remediation measures and plants established. It is known that soil microbial diversity is critically associated to soil functioning, among which the most obvious one is to support plants^2^. Thus, the extent and direction of microbial structural shift in tailings is not only an indicator for soil development but also an essential factor for plant sustainability even in a short-term. Yet we still know very little about how phytostabilization practice changes tailings microbial community structure and what edaphic factors in tailings control the changes under field conditions.

Depending on the ore type, tailings can be quite diverse in their physicochemical properties, but can generally be described as sandy/silty, oligotrophic and toxic^3^. In base metal mines, the tailings are always sulphidic and abundant in residue metals and metalloids (mainly As)^4^,^5^. These tailings are normally dumped as impoundments devoid of vegetation. To reduce the adverse effects of legacy tailings, surface stabilisation through revegetation (i.e. phytostabilization) is legally required in many parts of the world. However, in contrast to phytoremediation of contaminated land^6^ or mine spoils^7^, phytostabilization of sulphidic base metal tailings is highly constrained by the inability of the tailings to support plants.

Microbial communities are essential players in soil functioning^8^ and biogeochemical cycling of tailings^9^. In contrast to acidic mine environments, the diversity and structure of microbial communities in neutral/alkaline tailings has not been extensively studied. Therefore, there had been little understanding of the dynamics of microbial communities during long-term field phytostabilization trials in extensively weathered sulphidic tailings in alkaline pH condition. Significant microbial biomass can be present in the oxidized layer of neutralised base metal tailings, but with lower microbial diversity (mainly bacteria) compared to natural soils^9^,^10^,^11^,^12^. A limited number of studies have also implied that the establishment of pioneer plants in Pb-Zn tailings increased microbial biomass and changed the community structure^11^,^13^. Nevertheless, how the microbial community structure in neutral base metal tailings changes with organic matter amendment and pioneer plant colonization, and the edaphic factors that drive these changes, are yet to be explored.

We hypothesize that in weathered neutral base metal tailings organic matter addition and colonization of pioneer plant species could substantially increase microbial diversity and change the microbial community structure. This study took advantage of a long-term field trial established at a Pb-Zn-Cu mine tailings impoundment in northwest Queensland, under subtropical and semi-arid climatic conditions. Tailings samples were examined for microbial diversity through pyrosequencing using universal primers for the 16S rRNA gene and a detailed analysis of environmental variables. Specifically, we aim to 1) examine the microbial diversity in the tailings on both revegetated and un-revegetated sites and 2) explore the edaphic factors controlling the evolution of the microbial community structure in the tailings. Our results suggest that organic matter remediation and plant establishment could induce the succession of microbial communities in the weathered and neutral tailings. However we believe that this succession is suppressed by geochemical factors (e.g. soluble S, total Fe and total As), which are the products of weathering of primary minerals in the tailings; these factors are retained in tailings in the semi-arid climatic conditions, where leaching is limited.

## Results

The tailings were neutral (pH about 7), slightly/moderately saline (2–7 mS/cm; based on Australian classification), contained high levels of metals and metalloids (e.g. As, Zn), and low levels of nutrients (water soluble organic carbon (SOC) < 0.04%). SOC increased significantly in the woodchip amended and/or revegetated treatments, although other soil chemistries remained unchanged (Table 1).

There was a large heterogeneity in the physicochemistry among the replicates in all treatments due to long-term uneven watering, root distribution and salt movement (Supplementary Table 1). For example, in the T+P treatment, EC ranged from 2.1 mS/cm to 5.9 mS/cm, water content ranged from 1.1% to 13.0% and SOC from 19.2 mg/kg to 198.0 mg/kg. Such heterogeneity means that the 24 samples are unique to each other, thus expanding the gradient ranges of physicochemical conditions in the tailing samples. For ecological statistics, the number of samples in this study is treated as 24.

In total, 40,988 good quality reads were obtained in all the samples with pyrosequencing. These sequences were classified into 557 OTUs_0.97_, including five archaeal. The number of OTUs ranged from 78 (T+P_4) to 244 (T+P+W_4). The T+P+W treatment had significantly (*P* = 0.019, one-way ANOVA; below the same) higher OTU numbers than the other treatments. The Shannon index ranged from 3.90 (T+W_4) to 6.08 (T+P+W_2) across all the samples (Table 1). Again, the Shannon index was significantly (*P* = 0.007) higher in T+P+W than the other treatments.

Rarefaction analysis of the microbial communities is shown in Supplementary Figure 1. At a 3% genetic distance, all the Shannon indices tended to be saturated against the sampled sequence number. This indicated that, despite some samples (9 of 24) producing a fairly low number of quality reads (<1000), the pyrosequencing survey covered almost the full extent of the taxonomic diversity in all the tailings samples.

**Figure 1:**
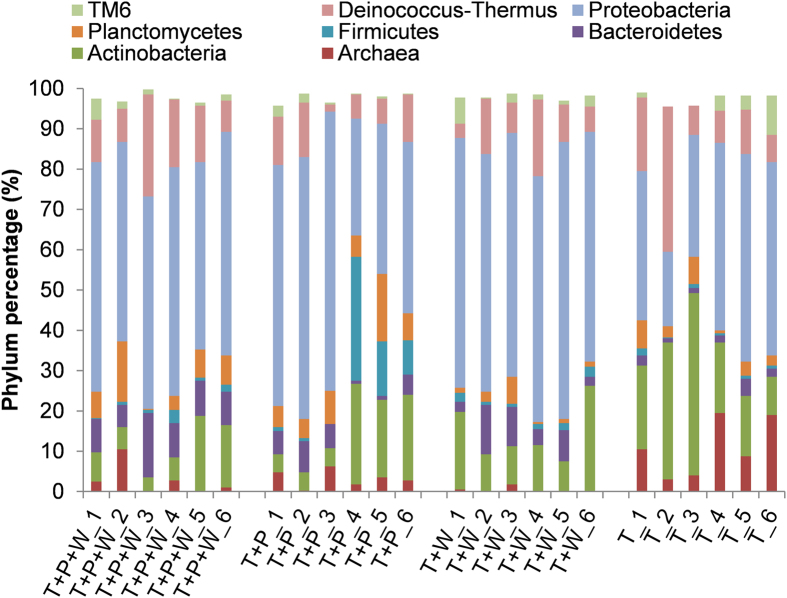
Phylum-level microbial composition of the tailings samples in this study. The phyla with a low frequency (<3%) in all the samples, including *Acidobacteria*, *Armatimonadetes*, *Chlamydiae*, *Cyanobacteria*, TM7, *SBR1093,* OP11 and *Chloroflexi*, were not shown in this figure.

The 554 bacterial and archaeal OTUs_0.97_ were affiliated with 18 phyla. Across all 24 samples, *Actinobac-teria*, *Bacteroidetes*, *Planctomycetes*, *Proteobacteria*, *Deinococcus-Thermus*, and *Crenarchaeota* were found to be the dominant phyla. Among these, *Actinobacteria*, *Proteobacteria* and *Deinococcus-Thermus* accounted for >60% of all the sequences, and for >72% in all the averaged treatments (Fig. 1).

The OTUs were further classified into 36 classes (6 OTUs_0.97_ were not assigned by the Classifier) and 57 orders (71 OTUs_0.97_ were not assigned). The dominant classes across all samples were *Alphaproteobacteria*, *Gammaproteobacteria*, and *Deinococci* (>1% in all samples). When averaged treatment data was used, eight classes were found to be >1% in all treatments.

**Figure 2:**
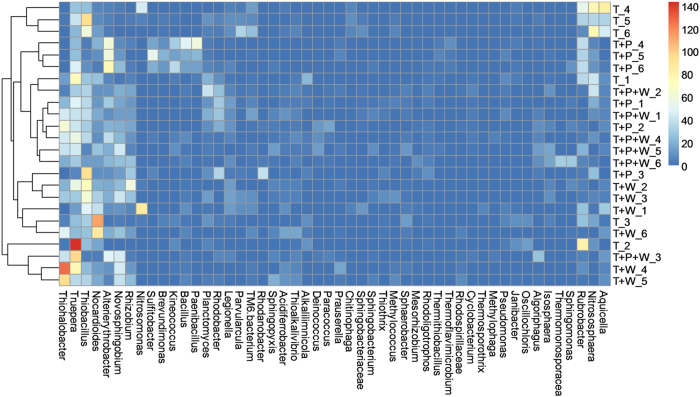
A heatmap showing the comparison and cluster analysis of microbial composition in the 24 tailings samples in this study. Indicator scores are based on the OTU abundance in the microbial assemblages. Only the OTUs with a frequency of >1% in at least 1 sample were shown in this figure.

Abundant OTUs in each sample were selected and a heatmap was generated for comparison among the samples (Fig. 2 and Supplementary Figure 2 for phylogeny of major OTUs). Interestingly, on average, only one genus, *Truepera*, was found to be >5% and four other genera (*Thiobacillus*, *Rubrobacter*, *Nocardioides* and *Altererythrobacter*) were >1% in all four treatments. When the 24 individual samples were compared, only the five genera listed above were present in all samples.

Due to the large variation among the replicates, the differences in abundance of most phyla were not statistically significant among the four treatments, with the exception of T+P+W, which was significantly higher in *Bacteroidetes* (*P* = 0.001, as above); the tailings-only sample harboured considerably higher Archaea (*P* < 0.001) and significantly lower *Proteobacteria* (*P* < 0.001) than the other treatments. At the genus level, T+W contained significantly higher in *Thiohalobacter* (*P* = 0.006); the two revegetated treatments contained less *Thiobacillus* (*P* < 0.04) and *Aquicella* (*P* < 0.02) than the two non-revegetated; T+P contained significantly higher *Altererythrobacter* sp. than the others (*P* < 0.001).

**Figure 3:**
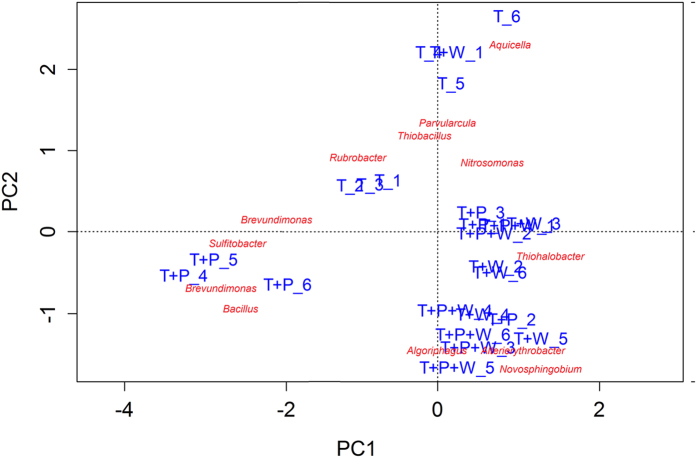
Biplots of the principal component analysis (PCA) at the replicate levels showing the correlation of genera with PCA axes. The PCA axes differentiate the tailings samples according to their microbial composition.

Microbial composition of the four treatments was differentiated by PCA at both the treatment (Supplementary Figure 3) and replicate levels (Fig. 3). The tailings-only treatment was clearly separated from the other treatments despite the substantial deviation among replicates. The tailings-only treatment was featured by a cluster of *Rubrobacter*, *Aquicella*, *Nitrososphaera*, *Parvularcula*, *Thiobacillus* and *Nitrosomonas*, while a cluster of *Altererythrobacter*, *Bacillus*, *Paenibacillus*, *Algoriphagus* and *Thermomonosporaceae* was dominant in the revegetated samples.

To explore which environmental factors best explain the microbial community structure, CCA was performed. A statistical analysis showed that significant correlations existed both between biological and environmental variables and among the biological or environmental variables (Supplementary Figure 4). Although HSOC was mainly controlled by SOC (r = 0.82) and SON (r = 0.43), the alpha diversity and Shannon index were both positively correlated with soluble Si (r = 0.63 and 0.60, respectively) and negatively correlated with soluble S (r = −0.28 and −0.31), total Fe (r = −0.31 and −0.51) and total As (r = −0.31 and −0.44).

**Figure 4:**
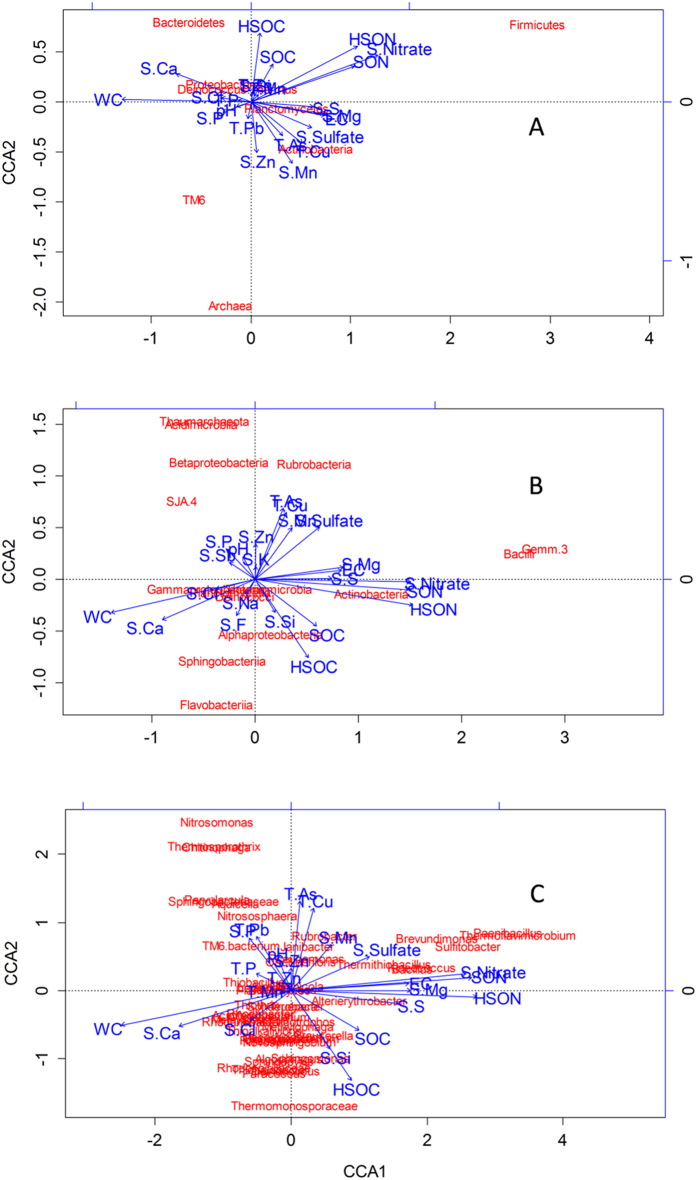
Biplots of canonical correspondence analysis of taxa abundance Vs. environmental variables in the 24 tailings samples. Three taxonomic levels, the phylum (**A**), the class (**B**) and the genus (**C**), were analysed. Phyla of maximum abundance <10%, classes of maximum abundance <3%, and genera of maximum abundance <1% in all the samples were excluded from the analysis. Abbreviations can be found in Table 1.

CCA results at the phylum, class and genus levels indicated that the soil N status and water content were the best predictors for microbial community structure (Fig. 4). Hot water soluble N, soluble N and water content explained 49%, 38% and 45% of the variation observed in the microbial community structure, respectively. Together, these variables explained up to 53% of the variation (Rank correlation method: Spearman; Resemblance measure: D1 Euclidean distance).

CCA further indicated that at the phylum level, *Firmicutes* was strongly associated with the soil N status (total soluble N, soluble NO_3_^–^ and hot water soluble N), Archaea with soil soluble Zn, and Mn and *Bacteroidetes* with soluble organic C. At the class level, *Rubrobacteria* were strongly associated with soluble Zn and soluble sulphate. *Bacilli* were strongly associated with the soil N pool. At the genus level, some prominent associations were also found. *Kineococcus*, *Paenibacillus* and *Bacillus* were strongly associated with soil N status and *Nitrososphaera* was associated with total As.

## Discussion

Structural shift of soil microbial communities under pressure from environmental factors is believed to have functional implications^14^. In the context of tailings revegetation, we expected that amendment and establishment of plants, even if only temporarily, could stimulate a change in the microbial community structure of the tailings towards a structure more similar to those of normal soils with sustainable biological capacity. Thus, exploring the factors controlling the microbial community structure in tailings in response to revegetation is important for assessing the efficacy of remediation measures and for gaining a better understanding of induced changes so superior remediation strategies can be developed.

As expected, the T+P+W treatment had significantly higher microbial diversity and biomass than the control, although the diversity and biomass in all treatments were still considerably lower than values reported for normal soils^15^,^16^. A substantial structural difference was observed between the control and treatments, with the emerging trend of increased organotroph-dominated microbes, compared to the lithotroph-dominated community in the tailings without amendment and plants. Yet surprisingly, soluble N, rather than SOC, was found to be the predominant factor driving the microbial structural evolution.

In the tailings used in this study, the concentrations of As, Cu, Pb and Zn were at least 101, 15, 362 and 23 fold higher, respectively, than the average crustal abundance^17^. Statistically, these elements are common stressors in Pb-Zn mine tailings worldwide (Supplementary Figure 5). High salinity is also of concern in the tailings, which would persist in the root zone profile due to the high geochemical reaction potential of tailings minerals and poor leaching in the semi-arid environment^3^. Though the tailings can be classified as moderately saline based on the data in this study, the pore water EC has reached values of up to 51.3 mS/cm over the past three years^3^. Moreover, nutrient deficiency is a common constraint for microorganisms living in tailings. An initial comparison (climatic factors were not considered) showed that the average SOC of the tailings samples was far less than the values reported for natural and arable soils^18^,^19^,^20^ and close to those of highly contaminated soils^21^.

Reflecting the stressors in this environment, diverse extremophiles were abundant in the tailings. At the treatment level, two potential extremophile genera, *Truepera* and *Rubrobacter*, were dominant and were shared by all treatments. The genera *Truepera*, within the *Deinococcales* group, and *Rubrobacter* affiliated to phylum *Actinobacteria*, both contain several well-characterized thermophilic radiation-tolerant species^22^,^23^,^24^,^25^. Due to the extreme DNA damage resistance inherent in these genera, they are possibly well-adapted to the oxidative conditions of the tailings caused by seasonal drought^26^, high levels of toxic elements^27^ and salinity^28^. Some other extremophiles, including the radiation-tolerant genera *Kineococcus* (>1% in eight samples)^29^ and *Deinococcus* (>1% in 11 samples)^30^ and a halophile genus *Thiohalobacter* (>1% in 15 samples)^31^, were also abundant in the tailings. These known radiation-tolerant, halophile, thermophile and/or metal-tolerant genera accounted for 27% (T+W_1) to 71% (T_2) (averagely 42%) of OTUs in all microbial assemblages.

A spectrum of potential S- and/or Fe-oxidizing genera was abundant in all samples. These included *Thiobacillus*^32^,^33^, *Thiohalobacter*^31^, *Sulfitobacter*^34^,^35^,^36^, *Acidiferrobacter*^37^, *Thioalkalivibrio*^38^,^39^,^40^, *Alkalilimnicola*^41^, and *Thiothrix* 42. These genera accounted for 6% to 42% (average 20%) of OTUs across the 24 microbial assemblages. The existence of abundant S- and/or Fe-oxidizing phylotypes using pyrites as a major energy source has been reported in many sulphidic tailings worldwide^9^,^43^,^44^,^45^. This again highlights the role of pyrites in shaping microbial communities associated with sulphidic tailings.

In terrestrial primary succession, higher plants only appear when soil properties reach a critical point^46^,^47^, with microbial diversity being one of the key soil properties^48^,^49^. Although abiotic factors pose significant constraints for plant establishment in mine tailings^50^, the development of microbial diversity is also crucial to facilitate and sustain plant growth and therefore must be part of the engineering efforts. Despite the overall low microbial biomass and alpha-diversity in all samples, this study found a substantial structural progression of microbial communities in response to phytostabilization (Supplementary Figure 3). Key changes from the tailings only to the revegetated treatments included the decrease of *Nitrososphaera*, *Rubrobacter*, TM6 bacteria and *Aquicella* and the increase of *Novosphingobium* and *Altererythrobacter*. The latter two genera are reported degraders of complex organic matter, for example, cellulose or aromatic compounds^51^,^52^,^53^. Further, the increase of heterotrophic species (mostly *Bacteroidetes* and *Proteobacteria*, e.g. *Rhizobium* and *Rhodobacter*) in the revegetated treatments was a major contributor to the increase in the Shannon index (data not shown). In natural soils, *Bacteroidetes* and *Proteobacteria* are among the most abundant phyla^54^ and are statistically associated with soil C turnover^55^. Many studies have shown that terrestrial microbial succession from non-vegetated to vegetated environments follows predictable patterns due to the convergence effects of plants^56^. Therefore, although mine tailings have a distinct initial microbial composition compared to other soil-formation materials like volcanic ash^57^ and glacier forefronts^58^,^59^, we speculate that phytostabilization may induce a directional change in the microbial community structure from autotroph- to heterotroph-dominant in sulphidic tailings, resembling natural primary succession^60^,^61^.

It is agreed in the literature that the driving factors for the evolution of a microbial community structure largely depend on the environmental gradient to which the communities are subjected or imposed under experimentally^62^. Organic C^63^, N availability^64^, pH^65^, salinity^66^,^67^, moisture^68^ and many other abiotic factors influence the microbial community structure in soil. Despite this, the edaphic gradients were not found to equally impact the community structure in the tailings of this study, with soluble N and water content being the best controllers. The predominant role of N in controlling the structure of the tailings microbial community is similar to findings in terrestrial primary succession, which indicated that N is a primary limiting factor at early stages of soil development^47^. In the tailings where SOC and SON were low and metal stress was high, *Nitrososphaera* were found to be dominant (Fig. 4A). Species within *Nitrososphaera*^69^,^70^ are typical ammonia-oxidizers well-adapted to a heavy metal-rich environment. They may have found a niche in the tailings and play a major role in ammonium-N oxidation. As significant nitrification needs sufficient ammonium-N^71^, the existence of abundant *Nitrososphaera* may also indicate active N fixation in these tailings samples, which was possibly performed by the other predominant genera *Rubrobacter* (CP000386 in Genbank) and *Truepera*^72^ that may harbour nitrogenases. *Firmicutes* were shown to respond positively to soluble N (Fig. 4A). Many genera within *Firmicutes,* such as *Paenibacillus* and *Bacillus*, are well-known degraders; some have been used as plant growth promoting rhizobacteria^73^. Thus, we found N availability to be a primary factor shaping the microbial communities associated with the tailings. For phytostabilization of the tailings, the addition of soluble N may shift the microbial community structure toward an increase in heterotrophs and could ‘prime’ nutrient cycling in the tailings.

It should be noted that all the associations discussed above are based on the premise that the microbial communities were shaped mainly by deterministic processes, specifically that habitat filtering effects are the dominant drivers in determining microbial composition in the tailings. Some random processes that are not accounted for here could also be important^74^. In this study, these may include competition among species in similar niches, as reflected by some of the negative associations detected among similar genera, and seed availability, which can be different among treatments by the introduction of plants or amendments. Meanwhile, once-off sampling may be not adequate to draw a reliable relationship between environmental factors and microbial community structure, since both of many factors, like water content and labile N, and microbial communities are dynamic under field conditions. Yet, as an extreme environment, sulphidic tailings may have strong selective effects on colonizing species, even if organic amendments and plant roots can create microhabitats, as shown in this study. To gain further understanding of the dynamic evolution of the microbial communities under the site conditions, it is useful to conduct repeated sampling from the same location in multiple seasons in the near future.

As expected, fairly low microbial biomass (i.e. hot water soluble carbon) and alpha-diversity (i.e. the number of OTUs_0.97_) were found in all the samples. This concurs with the ecological amplitude theory, which predicts that extreme habitats usually have low biomass and biodiversity relative to moderate habitats^14^,^75^,^76^. The average HSOC of the tailings in this study was less than 1/5 of the reported values for grassland and forest soils^20^,^77^. The number of microbial OTUs in the tailings obtained through in-depth tag-sequencing was close to those found in other tailings using 16S rDNA clone libraries. For example, around 160 OTUs_0.99_ were found at a Pb-Zn mine site in Graham County, Arizona, USA^43^; 23–50 OTUs_0.97_ were found in the Pb-Zn tailings in Yunnan, China^44^,^45^; and 86 OTUs_0.97_ were found in the Pb-Zn tailings in Guangdong, China^78^. For comparison, forest and grassland soils have considerably higher microbial alpha-diversity (around 3000 OTU_0.97_)^79^.

In contrast to natural pedogenesis, tailings contain a high abundance of stressors that may not be removed sufficiently and rapidly enough via natural succession^61^. In our semi-arid site, the removal of these stressors (soluble salts from mineral weathering) is exacerbated due to the lack of frequent water infiltration and very limited leaching processes. The negative role of pyrites (FeS_2_) in mine tailings in controlling microbial diversity has been indicated by the negative correlations between the alpha diversity plus Shannon index and total Fe, soluble S plus total As. While previous studies have confirmed that the tailings in this study are abundant in pyrites^80^, the soluble S can be an indicator of active pyrite weathering^81^,^82^. Active weathering of pyrite will release not only saline ions by dissolving carbonates, but also toxic elements, especially As, as various sulphides normally associate with pyrite as sulphide paragenesis^83^. Indeed, Fe was strongly correlated with total As (r = 0.73), total Cu (r = 0.66), total Mn (r = 0.78), total Zn (r = 0.54) and total Pb (r = 0.61) in this study. Meanwhile, as estimated in many studies^81^,^84^, pyrite oxidation can continue for hundreds of years in most pyrite-abundant tailings. From an ecological engineering perspective, this means that phytostabilization of tailings is not practical for creating a safe site for either belowground or aboveground communities due to the long-term release of saline or metal/metalloid elements. Therefore, the roles of pyrite-eaters, metal-tolerant and halophile genera in soil functioning merit further attention.

## Conclusions

Phytostabilization of sulphidic tailings remains a challenging enterprise. As indicated by the results presented in this study, the direct revegetation of tailings with amendment of woodchips induced significant increases in both microbial diversity and biomass and directed the microbial community structure towards one more similar to those of natural soils. Those changes, however, did not modify the tailings sufficiently to meet the requirements of a functioning soil. The low microbial diversity and biomass of the communities and the dominance of extremophiles in all the treatments means additional inputs and on-going improvement would be required, in addition to the initial amendment and direct revegetation with pioneer plant species, although the addition of N fertilizers and a water regime could be used to ‘manipulate’ microbial communities. Considering that rapid removal of most stressors in tailings via leaching is not a practical option under semi-arid climatic conditions, searching for more efficient amendments to ameliorate the tailings is a priority for the phytostabilization of tailings^85^,^86^.

## Materials and Methods

### Sampling

The field trial site was located at a Pb-Zn-Cu mine tailings impoundment in northwest Queensland, with a subtropical and semi-arid climate with intensive rainfall in summer. The mine had been in operation as a Pb-Zn-Ag and/or Cu mine for over 80 years and was decommissioned about 30 years ago. Accumulated mine tailings in this area were estimated to be over 100 million tonnes^80^.

The field trial was established in 2010 to investigate options for direct revegetation of the tailings, which had experienced 40 years of natural weathering. Briefly, four treatments were designed as follows: tailings-only as control (T), tailings directly revegetated with native plants (T+P) (see below), tailings with woodchip amendment (T+W) and tailings with woodchip amendment and directly revegetated with native plants (T+P+W). The woodchips were woody mulch from local trees. A total of 2 m^2^ plots were established for each treatment. In the treatments amended with woodchips, 20% (v:v) of the total volume of woodchips was amended into the tailings. Plants established in the tailing treatments were mixed native species (with 5–6 plants each species), including *Acacia chisolmii*, *A. ligulata*, *Atriplex nummularia*, and *Ptilotus exaltatus*. These were transplanted as tubestock seedlings. More information about treatment and plant status can be found in Supplementary Table 1. The field trial was initially established to compare main effects of these amendment approaches, but extreme field conditions resulted in high variability even within treatments. As a result, the data from subsamples were pooled for correlation analysis rather than simple treatment comparison, to explore major edaphic factors associated with microbial community profile.

Tailings were sampled in June 2013. Six separate subsamples were taken at six randomly distributed spots across the 2 m^2^ surface area of each treatment. This sampling method resulted in six quasi-replicates for each treatment. And considering the environmental heterogeneity in the field (as characterized by the physiochemistry of the samples below), the 24 subsamples have been treated ecologically as separate sample units for statistical analyses, in a similar manner as a survey. Samples were placed into double sealed plastic bags in a pre-cooled icebox (<10 °C) and shipped to the laboratory within 24 h. During DNA extraction, samples were stored in a cold room (4 °C) and were extracted within eight days. Aliquots of each replicate subsample were oven-dried for physiochemical analyses.

### Physicochemistry

For total elemental analysis, ball-milled tailings samples were digested with a 5:3:2 solution of nitric, hydrochloric and hydrofluoric acids using a microwave system^87^. Total elements after digestion were then analysed using ICP-AES. Water soluble elements were extracted by shaking 1 g sieved tailings (2 mm) in 50 ml DI water for 1 h at room temperature^4^. The elements in the water extracts were then determined using ICP-AES after filtering through a 0.45 μm filter. pH and electrical conductivity (EC) were determined electrically with a soil/water ratio of 1:5. Soluble anions in the water extracts were determined using an ion chromatograph DX-120 (Dionex Corporation) equipped with Column AS-22. Water and hot water soluble C and N were analysed following the methods described by Ghani *et al.*^77^. Briefly, 3 g dry tailings were shaken with 30 ml distilled water for 30 min at room temperature. After centrifugation at 3500 rpm for 20 min, the supernatant was filtered through 0.45 μm filter for water soluble C and N analyses. A further 30 ml of water was added to the sediments and the tubes were shaken for 16 h in a water bath at 80 °C. Following further centrifugation, the supernatant was collected as described above for C and N analyses. C and N in the solutions were determined using a Shimadzu Total Organic Carbon Analyser with Total Nitrogen detector. Hot water soluble organic C was used as a proxy of microbial biomass^77^.

### DNA extraction

DNA extraction from the tailings samples was performed following the protocol established in our previous study^88^ with minor modifications. Briefly, 5 g of tailings together with 25 ml 0.2% sodium pyro-phosphate solution (pH 7.5) were added into a 50 ml Falcon^®^ tube and sonicated (2 × 30 s at moderate power output). The suspensions were immediately layered into a sucrose solution (25 ml of 1.3 g ml^−1^) in another 50 ml Falcon® tube, then centrifuged at 5,500 × g for 2 min. The clear upper sucrose fraction containing the microbial cells was poured into a new 50 ml Falcon® tube and centrifuged at 20,000 × g for 10 min at 4 °C. The top 1/3 supernatant was discarded and then the same volume of 0.8% NaCl solution was added to the tube and concentrated by centrifugation (20,000 × g for 10 min) at 4 °C. After discarding the supernatant, the cells plus fine particulates at the bottom of the tube were carefully rinsed with bead solution and transferred into the Bead Tube of PowerLyzer^TM^ PowerSoil^®^ DNA Isolation Kit (MO BIO Laboratories, Inc.) for DNA extraction.

The concentration and purity of the extracted DNA was determined using a Nanodrop spectrometer (Thermo Scientific, US). Quality DNA was submitted to the Australian Centre for Ecogenomics (ACE) within The University of Queensland for Pyrosequencing, using universal fusion primers 926F and 1392wR as described elsewhere^89^.

### Pyrosequencing data analysis

Pyrosequencing-derived data was analysed through the ACE pyrotag pipeline^90^. Briefly, after the data were ordered using QIIME^91^, the sequence reads were trimmed to 250 bp lengths and de-noised using ACACIA^92^. Sequences with 97% similarity were assigned to operational taxonomic units (OTUs_0.97_) by CD-HIT-OTU^93^ and aligned by Pynast^91^. A non-normalized OTU table and rarefaction curves were then generated by QIIME. A centroid normalized OTU table was generated using Nomaliser^94^. The normalized OTU table was used for further analyses.

### Statistics

A heatmap showing the abundance of major genera was generated using the package ‘pheatmap’^95^ in R version 2.15.1. These OTU sequences and reference sequences from Genbank were aligned using SINA^96^. The aligned sequences were then used to generate a neighbour-joining phylogenetic tree using MEGA 5^97^.

Linear correlation analysis (LCA), principal component analysis (PCA) and canonical correspondence analysis (CCA) were performed between the environmental variables and biological composition using R with the ‘vegan’ package^98^.

## Additional Information

**How to cite this article**: Li, X. *et al.* From lithotroph- to organotroph-dominant: directional shift of microbial community in sulphidic tailings during phytostabilization. *Sci. Rep.* **5**, 12978; doi: 10.1038/srep12978 (2015).

## Supplementary Material

Supplementary Information

## Figures and Tables

**Table 1:** Selected properties of the 24 tailings samples used in this study.

Treatment	Replicate and Code	Shannon index	OTUs number	EC (mS/cm)	pH	WC (%)	SOC	SON	HSOC	HSON	S-Sulfate	S-Nitrate (mg/kg)	S-P	S-Zn	T-As	T-Cu	T-P	T-Pb	T-Zn
Tailings only	T_1	5.49	107	5.62	6.9	4.27	19.97	2.83	40.80	1.49	20870	0	3.51	13.63	217.2	1037	160.4	3620	1816
	T_2	4.28	123	6.68	6.9	2.66	37.47	3.86	44.54	1.52	29347	1.04	1.85	18.01	301.9	1230	230.4	5930	2910
	T_3	5.02	166	4.70	6.7	4.50	8.96	2.09	31.25	1.15	16935	1.05	3.12	12.72	266.5	1464	233.9	5250	2532
	T_4	3.94	92	2.26	6.8	12.73	11.11	2.22	38.16	1.23	13513	0	5.58	11.00	320.6	1312	319.3	6760	3224
	T_5	5.07	111	3.22	7.4	12.79	11.37	2.43	40.20	1.46	11716	1.03	3.12	12.38	303.9	1382	330.8	6730	3409
	T_6	4.68	105	2.89	6.8	12.63	23.24	2.49	40.09	1.23	8655	0.38	1.76	11.55	309.9	1253	259.3	6400	3134
Tailings revegetated	T+P_1	5.93	149	5.42	6.8	9.41	197.91	11.19	130.21	9.37	17521	6.11	1.67	9.42	286.3	1272	238.9	5300	3234
	T+P_2	5.67	200	2.59	6.9	12.93	43.58	11.49	72.71	5.83	14340	10.85	3.87	4.15	299.7	1334	330.1	6370	3579
	T+P_3	5.14	95	2.08	6.9	9.85	19.18	6.92	78.14	5.83	19352	5.67	1.93	13.33	275.0	1275	308.9	5090	3164
	T+P_4	4.71	78	4.59	6.8	1.07	49.27	38.99	74.01	20.27	15982	42.89	1.65	8.73	305.4	1308	281.7	5660	3275
	T+P_5	5.56	181	5.89	6.9	1.15	95.97	56.79	83.65	21.85	27719	17.74	2.63	11.59	299.3	1299	254.8	5910	3300
	T+P_6	4.91	84	5.51	6.9	1.42	78.60	62.10	86.78	17.39	14008	22.91	3.79	16.00	268.8	1249	249.6	5290	2938
Tailings with woodchips amendment	T+W_1	4.79	101	2.61	6.9	11.34	12.84	1.77	46.93	1.54	10357	0.40	7.06	8.79	296.4	1227	280.3	6490	3070
	T+W_2	4.94	107	3.12	6.7	10.97	53.46	4.24	74.81	4.30	9659	0	3.33	9.58	286.8	1255	265.7	6350	2991
	T+W_3	5.29	139	2.86	6.9	10.26	22.14	2.71	61.24	3.05	14330	0	0.84	7.88	316.3	1260	265.5	6410	3192
	T+W_4	3.89	98	3.26	6.9	9.87	29.76	3.16	53.01	1.88	10328	0	2.49	17.49	297.8	1114	265.2	6000	3287
	T+W_5	4.94	132	3.04	6.8	10.65	32.89	4.25	53.59	3.12	18580	11.18	2.76	11.27	258.6	1166	260.0	5860	3261
	T+W_6	4.82	124	3.39	6.8	11.04	20.67	3.18	74.50	3.52	12530	0.45	1.88	5.25	259.8	1334	334.7	6140	3485
Revegetated tailings with woodchips amendment	T+P+W_1	5.57	162	3.90	6.9	12.87	36.83	6.99	83.73	5.67	9582	0.88	4.86	5.48	213.2	1086	215.8	4200	2409
	T+P+W_2	6.08	150	5.49	6.7	11.42	82.69	14.64	96.33	5.96	14943	1.63	3.53	37.54	240.8	1052	322.6	5080	3914
	T+P+W_3	4.54	138	3.86	6.9	13.00	45.21	9.04	71.69	5.87	10999	1.09	2.41	5.27	237.3	1029	236.0	4700	2620
	T+P+W_4	5.89	244	3.86	6.8	10.31	29.50	22.67	77.03	11.78	5628	0	6.61	4.09	224.9	1144	259.8	4930	2589
	T+P+W_5	5.83	212	3.81	6.7	8.76	47.91	8.19	69.10	5.34	12247	1.95	3.74	15.27	284.9	1240	315.6	6300	3189
	T+P+W_6	6.03	133	2.99	7.0	11.34	47.95	11.69	83.91	6.12	6595	3.30	4.00	8.30	258.9	1046	250.9	5890	2772

Notes: WC, water content; EC, electrical conductivity; SOC, soluble organic carbon content; SON, soluble nitrogen content; HSOC, hot water soluble organic carbon content; HSON, hot water soluble nitrogen content; “S” and “T” as prefixes with elements/ions stand for soluble and total, respectively.
