# Supplementary material for: From lithotroph- to organotroph-dominant: directional shift of microbial community in sulphidic tailings during phytostabilization
Source: Sci Rep. 2015 Aug 13;5:12978. doi: 10.1038/srep12978 (PMC4534789; doi:10.1038/srep12978)
Supplement: Supplementary Information [file srep12978-s1.pdf]

From lithotroph- to organotroph-dominant: directional shift of microbial community in sulphidic tailings during phytostabilization

Xiaofang Li[1], Philip L. Bond[2], Joy D. Van Nostrand[3], Jizhong Zhou[3,4,5*] and Longbin Huang[1*]

[1] *Centre for Mined Land Rehabilitation, Sustainable Minerals Institute, The University of Queensland, QLD 4072, Australia*

[2] *Advanced Water Management Centre, The University of Queensland, QLD 4072, Australia*

[3] *Institute for Environmental Genomics and Department of Microbiology and Plant Biology, University of Oklahoma, Norman, OK 73019*

[4]*Earth Science Division, Lawrence Berkeley National Laboratory, Berkeley, CA, USA 94720*

[5]*State Key Joint Laboratory of Environment Simulation and Pollution Control, School of Environment, Tsinghua University, Beijing 100084, China*

Running title: Microbial communities in sulphidic tailings

Subject Category: Microbial population and community ecology

*Corresponding authors: l.huang@uq.edu.au; jzhou@rccc.ou.edu

Supplementary Table 1 Additional information of the treatments sampled in this study

Treatment name	Top soil addition	Plant status	Watering
Tailings only	No topsoil was added initially	No plants	Salt on surface
Tailings + plants	Surfaced with local topsoil (gravelly/siliceous soil) 10-15 cm thick	Surviving plants include 2 x Ptilotus, 3x Acacia chisolmii, 1 x Acacia ligulata	Irrigation
Tailings + Woodchips	No topsoil was added initially	No plants	Salt on surface
Tailings + Woodchips + plants	No topsoil was added initially	4 x Ptilotus plants, 2 x Acacia chisolmii plants alive	Drip-irrigation was not uniformly distributed

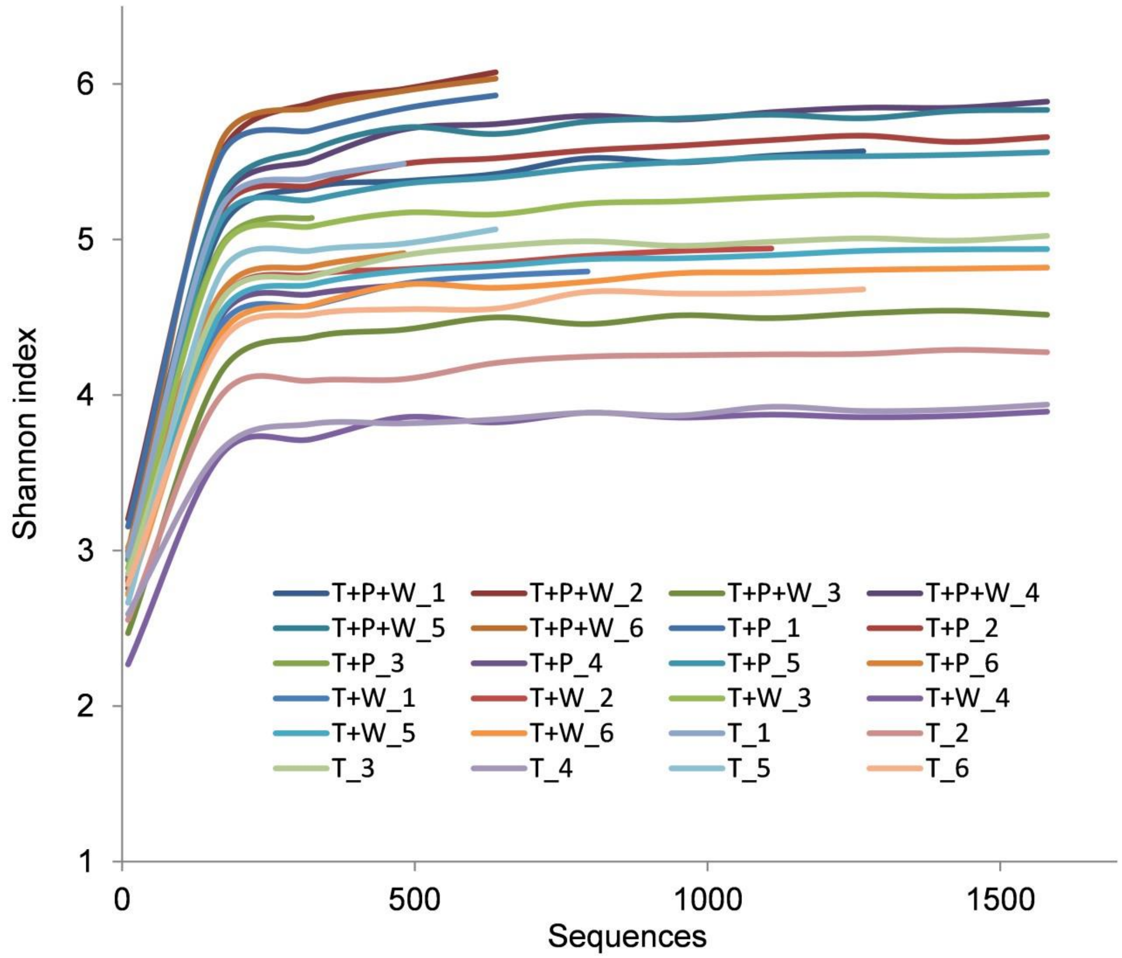

Supplementary Figure 1 Rarefaction curve of OTUs recovered from the Mount Isa Pb-Zn-Cu tailings

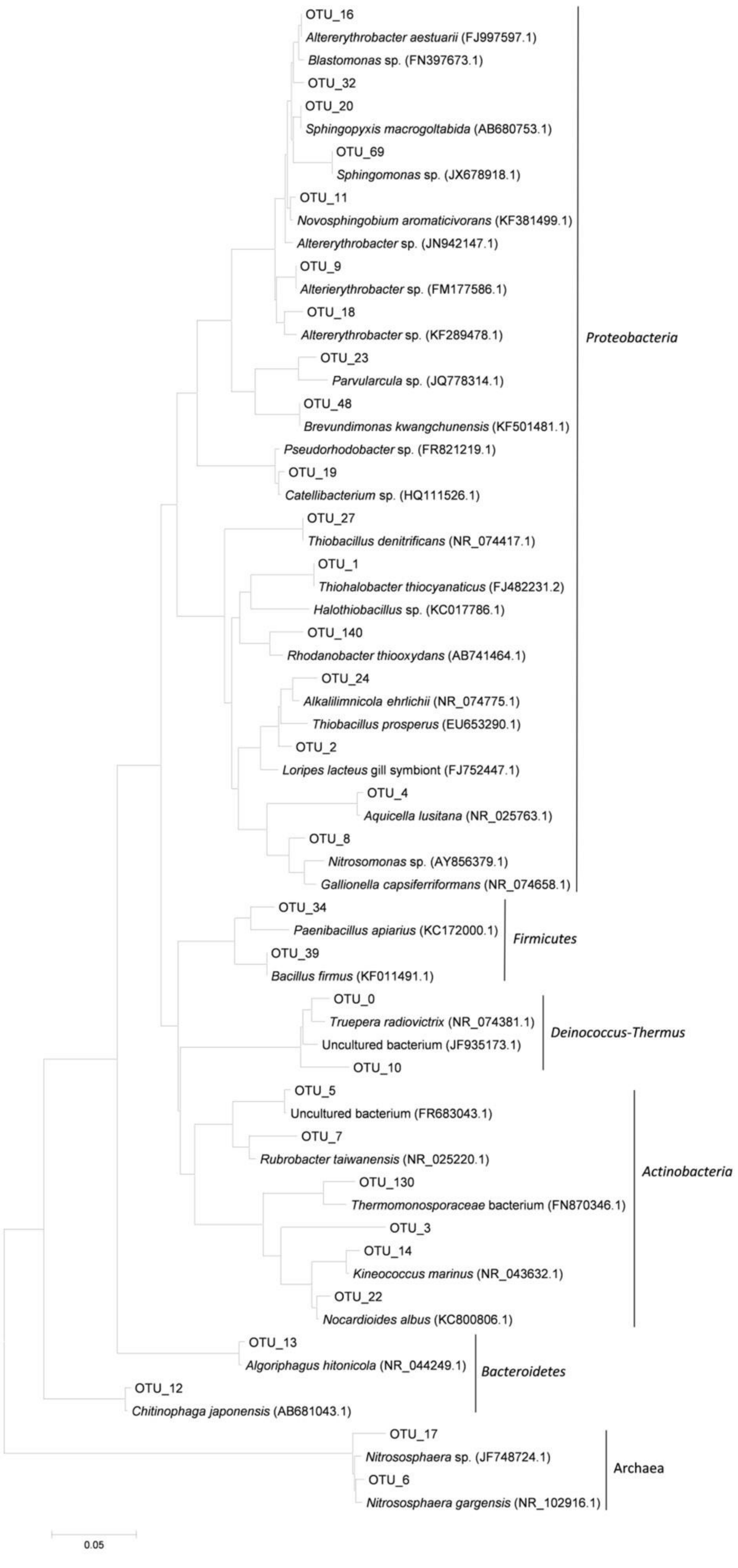
OTU_16
Altererythrobacter aestuarii (FJ997597.1)
Blastomonas sp. (FN397673.1)
OTU_32
OTU_20
Sphingopyxis macrogoltabida (AB680753.1)
OTU_69
Sphingomonas sp. (JX678918.1)
OTU_11
Novosphingobium aromaticivorans (KF381499.1)
Altererythrobacter sp. (JN942147.1)
OTU_9
Alterierythrobacter sp. (FM177586.1)
OTU_18
Altererythrobacter sp. (KF289478.1)
OTU_23
Parvularcula sp. (JQ778314.1)
OTU_48
Brevundimonas kwangchunensis (KF501481.1)
Pseudorhodobacter sp. (FR821219.1)
OTU_19
Catellibacterium sp. (HQ111526.1)
OTU_27
Thiobacillus denitrificans (NR_074417.1)
OTU_1
Thiohalobacter thiocyanaticus (FJ482231.2)
Halothiobacillus sp. (KC017786.1)
OTU_140
Rhodanobacter thiooxydans (AB741464.1)
OTU_24
Alkalilimnicola ehrlichii (NR_074775.1)
Thiobacillus prosperus (EU653290.1)
OTU_2
Loripes lacteus gill symbiont (FJ752447.1)
OTU_4
Aquicella lusitana (NR_025763.1)
OTU_8
Nitrosomonas sp. (AY856379.1)
Gallionella capsiferriformans (NR_074658.1)
Proteobacteria
OTU_34
Paenibacillus apiarius (KC172000.1)
OTU_39
Bacillus firmus (KF011491.1)
Firmicutes
OTU_0
Truepera radiovictrix (NR_074381.1)
Uncultured bacterium (JF935173.1)
OTU_10
Deinococcus-Thermus
OTU_5
Uncultured bacterium (FR683043.1)
OTU_7
Rubrobacter taiwanensis (NR_025220.1)
OTU_130
Thermomonosporaceae bacterium (FN870346.1)
OTU_3
OTU_14
Kineococcus marinus (NR_043632.1)
OTU_22
Nocardioides albus (KC800806.1)
Actinobacteria
OTU_13
Algoriphagus hitonicola (NR_044249.1)
OTU_12
Chitinophaga japonensis (AB681043.1)
Bacteroidetes
OTU_17
Nitrososphaera sp. (JF748724.1)
OTU_6
Nitrososphaera gargensis (NR_102916.1)
Archaea
0.05

Supplementary Figure 2 A phylogenetic tree of 16S rRNA gene sequences recovered from the 24 tailings samples in this study. The tree was constructed using the neighbour-joining method. Only representative OTUs were shown in this tree. The taxonomic groups are delineated on the right. Scale bar stands for 0.05 changes per site

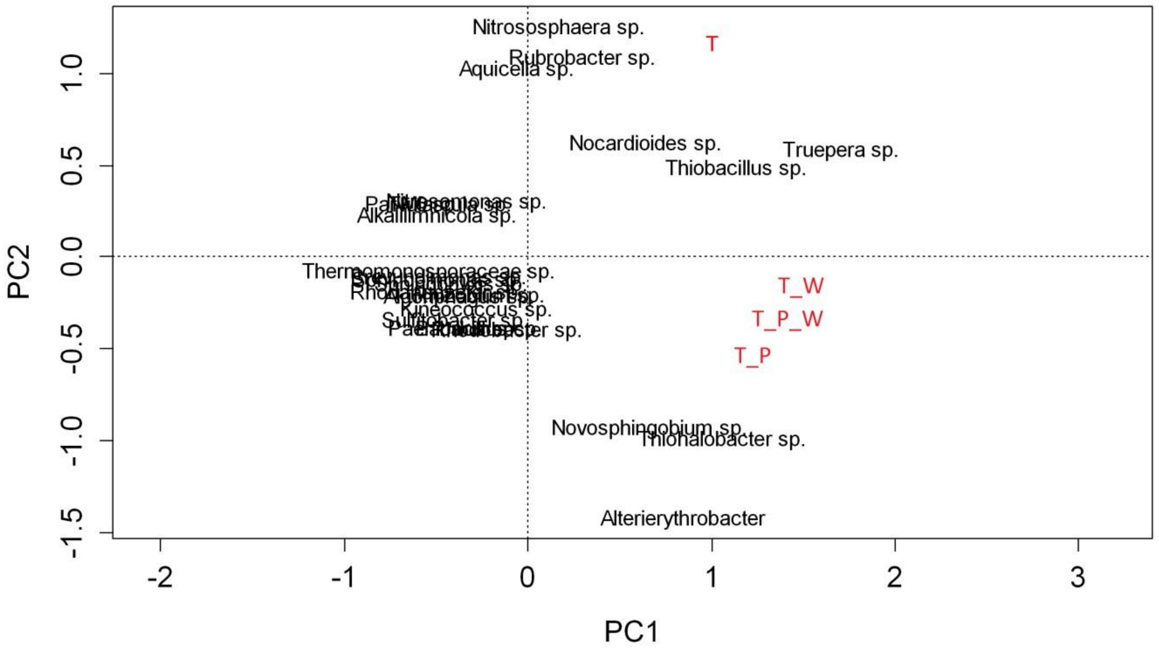

Supplementary Figure 3 Biplots of the principal component analysis (PCA) at the treatment level showing the correlation of genera with PCA axes. The PCA axes differentiate the tailings samples according to their microbial composition

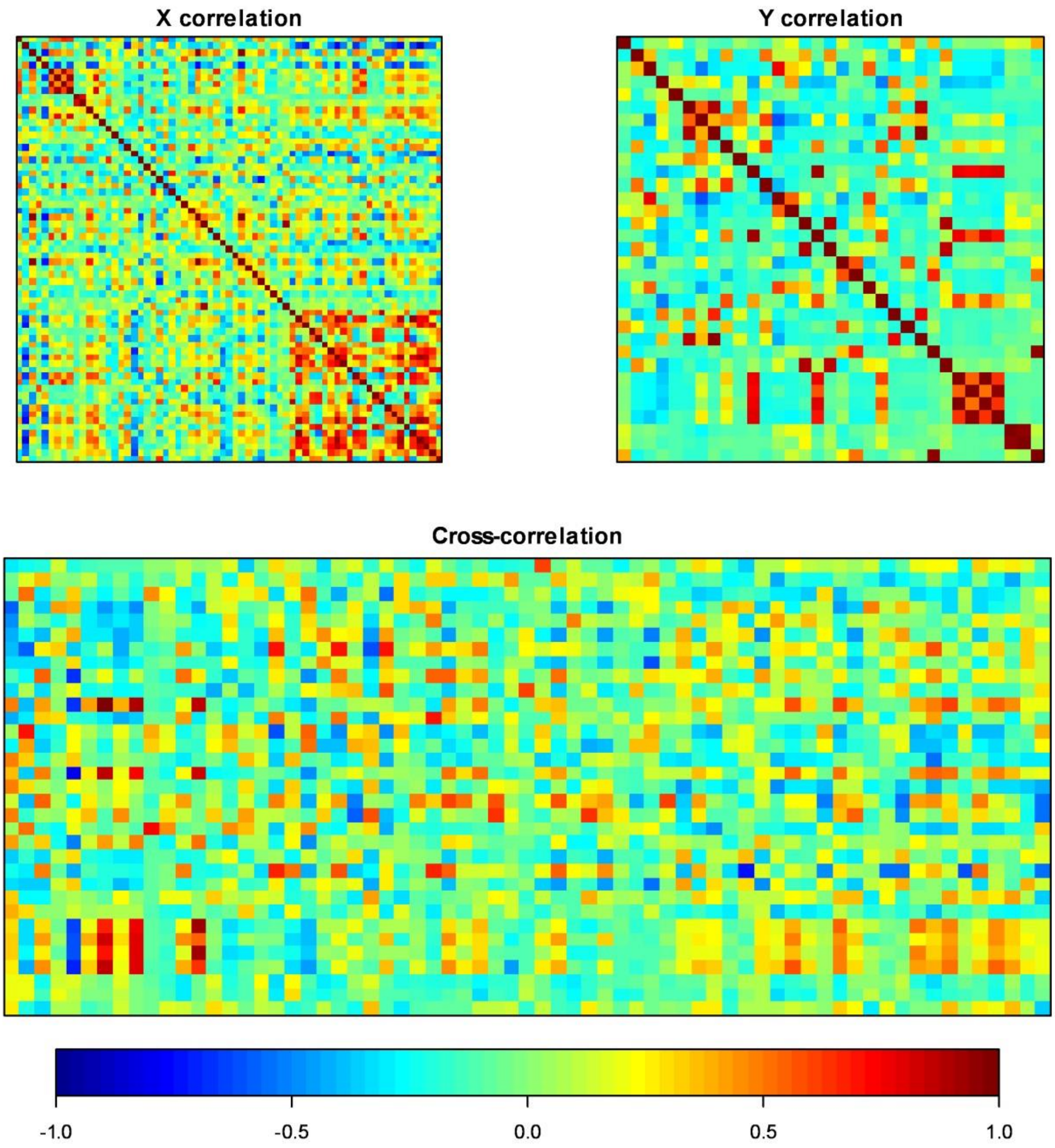

Supplementary Figure 4 Correlation matrices for: Environmental variables (upper-left, 67 × 67), biological variables (upper-right, 33 × 33), cross-correlation Environmental _ biological variables (bottom). Increasing values are translated into colours from blue (negative correlation) to red (positive correlation)

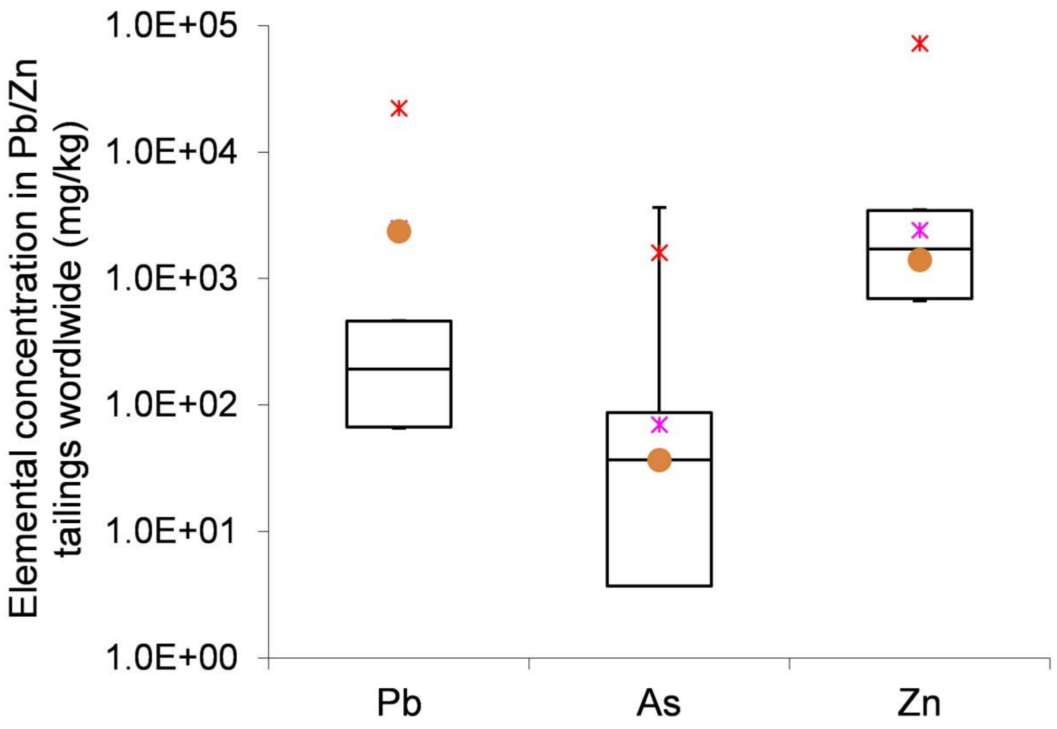

Supplementary Figure 5 Abundance of prominent toxic elements (Pb, n = 52; As, n = 38; Zn, n =5 4) in Pb-Zn mine tailings from worldwide. Data was from a Tailings Property Database constructed by our group (available for free upon request)
